# Clinicopathological and prognostic significance of long non-coding RNA EWSAT1 in human cancers: A review and meta analysis

**DOI:** 10.1371/journal.pone.0265264

**Published:** 2022-03-14

**Authors:** Jian Wen, Haima Li, Dongdong Li, Xieping Dong

**Affiliations:** 1 People’s Clinical Medical College affiliated to Nanchang University, Nanchang, Jiangxi, China; 2 Department of Orthopedics, Jiangxi Provincial People’s Hospital, Nanchang, Jiangxi, China; 3 Department of Neurosurgery, Jiangxi Provincial People’s Hospital, Nanchang, Jiangxi, China; 4 Department of Pulmonary and Critical Care Medicine, Jiangxi Provincial People’s Hospital, Nanchang, Jiangxi, China; Universita degli Studi della Campania Luigi Vanvitelli, ITALY

## Abstract

**Background:**

Ewing sarcoma-associated transcript 1 (lncRNA EWSAT1) is reported to have a close relationship with the overall survival in many cancers. However, the role of its prognosis and correlations with the clinicopathological features in different cancers haven’t been explored yet. Herein, we intend to assess the prognostic value and correlations with the clinicopathological features in several cancers.

**Methods:**

PubMed, Embase, Web of Science, and The Cochrane Library were searched for literature review from inception to October 25, 2021. Valid data was extracted to make forest and sensitivity analysis plots using Review Manager 5.4 and Stata software. Hazard ratio (HR) or odds ratio (OR) with 95% confidence interval (CI) was used to evaluate the relationship between different expression of EWSAT1 and patients’ prognosis and clinicopathological features.

**Results:**

7 studies were screened for this review, including 550 samples. Meta-analysis showed that high expression of lncRNA EWSAT1 was associated with poor overall survival (OS) (HR = 2.10, 95% CI, 1.60–2.75, *p* < 0.0001) in cancers reported. In addition, patients in high expression group of EWAST1 tended to have more metastasis (OR = 2.20, 95% CI 1.47–3.31, *p* = 0.0001), and higher TNM stage (I+II vs. III: OR = 0.34, 95% CI 0.21–0.56, *p* < 0.0001), but in the same time with higher differentiation (well + moderate vs. Poor: OR = 2.21, 95% CI 1.02–4.76, *p* = 0.04). Age (OR = 1.47, 95% CI 0.94–2.30, *p* = 0.09) was not significantly different in patients with aberrant expression of EWSAT1.

**Conclusions:**

Our study shows that high expression of EWSAT1 may indicate poor overall survival and associated with several clinicopathological features, which can be used as a potential prognosis biomarker for multiple cancers.

## Introduction

Cancer is a worldwide health problem, which usually result in high cost, high difficulty to treat and high mortality rate [1]. Although great progresses have been made in early diagnosis and individualized treatment, yet we have to admit that there’s still no breakthrough in this area [2, 3]. More knowledge is expected to further elucidate how cancers generate, proliferate, progress and metastasize, how they interact with clinicopathological features, treatment and prognosis. Functions of non-coding RNAs are exactly one of the most important keys to this problem [4, 5].

Long non-coding RNAs undertake many important and complex functions in human body, like regulating transcription, splicing pre-mRNA, and acting as molecular sponges and scaffolds, etc. Many studies show aberrant expression of lncRNA can lead to various dysfunctions of human body, including cancers [6]. It’s necessary and urgent to explore the relationship between lncRNA and cancers, which may unveil potential approaches for early diagnosis, cure and prognosis of cancers [7, 8].

Ewing sarcoma-associated transcript 1 (lncRNA EWSAT1), located on human chromosome 15q23, only high expression in normal human testis and kidney, was recently reported more and more frequently to have intimate association with many different cancers, such as Ewing carcinoma [9], lung cancer [10], nasopharyngeal carcinoma [11], osteosarcoma [12–14], cervical cancer [15], colorectal cancer [16, 17], ovarian cancer [18], glioma [19]. And aberrant expression of EWSAT1 was also said to affect the OS of patients in some of the cancers above. Thereby, this meta-analysis intended to explore the overall effect of aberrant EWSAT1 expression with OS in cancers and bridge gap in knowledge between aberrant expression of EWSAT1 and the prognosis and clinicopathological features of patients in different kinds of cancers.

## Materials and methods

### Registration

The study was registered on PROSPERO (the registration number is: CRD42021289394).

### Search strategy

Comprehensive searches of PubMed, EMBASE, Web of Science and the Cochrane Library were performed by two independent researchers restricted to English language articles from inception to October 25, 2021. The search terms were as follows: (“EWSAT1” OR “RNA-277” OR “TME84” OR “LINC00277” OR “NCRNA00277” OR “Ewing sarcoma-associated transcript 1” OR “long non-coding RNA EWSAT1” OR “lncRNA EWSAT1”).

### Inclusion and exclusion criteria

The inclusion criteria were as follows: (1) Cancers with aberrant expression of lncRNA EWSAT1; (2) patients were divided into two groups based on lncRNA EWSAT1 expression levels; (3) the study provided at least one of the following clinical outcomes: age, OS or K-M curve (sufficient data to compute the hazard ratio (HR) and 95% CI of survival), histological (differentiation) grade, TNM stage, distant/lymphnode metastasis. The exclusion criteria were as follows: (1) publication letters, case reports, reviews, conference abstracts, etc.; (2) studies without or not enough clinical data; (3) studies unrelated to lncRNA EWSAT1.

### Data extraction

All articles were reviewed by two independent well trained investigators and a third author was referred when there was any disagreement. The following information was collected according to the inclusion criteria: name of first author, publication year, country of origin, tumor type, sample type, sample size, detection method, Hazard ratio (HR) of OS between high and low EWSAT1 expression groups and the corresponding 95% CI (if not provided directly while a Kaplan-Meier curve was available, Engauge Digitizer version 4.1 and Tierney’s method were used to extract HR, 95% CI, Variance and O-E), and the quality of each study was assessed by Newcastle-Ottawa Scale (NOS).

### Quality assessment

The quality of the literature was evaluated by Newcastle-Ottawa Scale (NOS) with a total score of 10. The higher the score, the better the quality of the literature.

### Data synthesis and statistical analysis

When there was only a Kaplan-Meier curve available, we would use Engauge Digitizer version 4.1 and Tierney’s method to calculate HR, 95% CI, Variance and O-E. Then, Review Manager 5.4 was employed for further data analysis. Q-test and *I*^*2*^ were applied to evaluate the heterogeneity among the studies. If the heterogeneity was substantial (*P* < 0.10, *I*^*2*^ > 50%), the random effect model would be adopted, otherwise the fixed effect model would be used. Forest plots were drawn to display the meta-analysis results and funnel plots were used to assess any prospective bias in the publications. Sensitive analyses were conducted using local sensitivity analysis approach by excluding each eligible study one at a time, with plots drawn by Stata 12.0 (Stata, College Station, TX, USA). Finally, *P* < 0.05 was considered statistically significant in our analyses above.

### Expressions of EWSAT1 in pancancer

The TCGA PanCancer Atlas refers to a data set of molecular and clinical information from over 10,000 tumors representing 33 types of cancer [20]. Therefore, UCSC Toil RNA-seq Recompute data of TCGA and GTEx database (https://xenabrowser.net/datapages/) was used to explore the expression of EWSAT1 in pancancer (33 types of cancer in TCGA) by R3.6.3 [21]. Wilcoxon rank sum test was applied to detect the differences between normal and tumor groups in pancancer. And ggplot2 package in R was adopted for result visualization.

## Results

### Characteristics of studies

**Fig 1:**
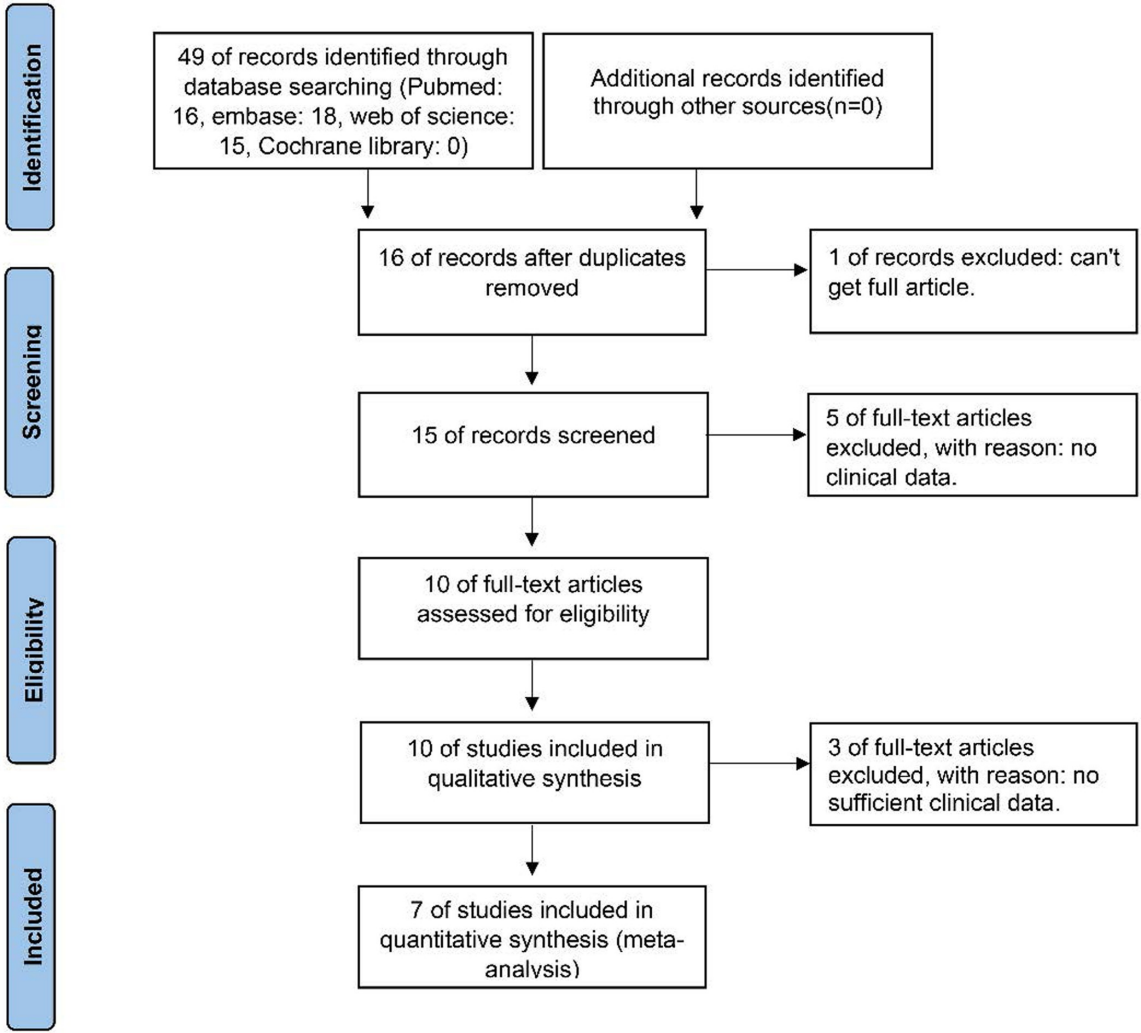
Flow diagram of this meta-analysis.

In this meta-analysis, 7 studies (Peng Song, etc. 2016; G.-Y. ZHANG, etc. 2017; Iulia Virginia Iancu, etc. 2017; R. ZHANG, etc. 2018; HUI YANG, etc. 2020; Jing Liu, etc. 2021; Dewei Shen, etc. 2021) were included for meta-analysis after screening 49 candidate articles. Fig 1 shows our screening strategy and results, according to the PRISMA guidelines.

This study involved 550 patient form China (6 studies, n = 527) and Romania (1 study, n = 23), including 4 cancers: cervical cancer (n = 23), colorectal cancer (n = 151), glioma (n = 42), nasopharyngeal carcinoma (n = 108), osteosarcoma (n = 226). The minimum sample size of each study was 23, and the maximum was 176. Publication Time started from 2016 to 2021. The expressions of EWSAT1 were all detected by qRT-PCR with cancer tissues. The cutoff value for dividing high and low expression group of EWSAT1 varied in the studies (Table 1). The HRs and 95% CIs of OS between high and low expression groups of EWSAT1 were all extracted from Kaplan-Meier curves provided by original articles. 3 out of 7 articles got a 7 score of NOS and 4 got 6. The main essential characteristics of these studies are shown in Table 1.

**Table 1:** Main characteristics of the included studies.

Cancer	First author	Year	Country	Sample type	Sample size(n)	Detection method	Cutoff	HR(95%CI) of OS	NOS
nasopharyngeal carcinoma [22]	Peng Song	2016	China	tissue	108	qRT-PCR	2.36 Foldchange	2.25(0.97–5.20)	6
osteosarcoma [12]	G.-Y. ZHANG	2017	China	tissue	176	qRT-PCR	mean	2.02(1.27–3.21)	7
cervical cancer [15]	Iulia Virginia Iancu	2017	Romania	tissue	23	qRT-PCR	median	NA	6
colorectal cancer [16]	R. ZHANG	2018	China	tissue	106	qRT-PCR	Not mentioned	1.64(0.97–2.77)	6
glioma [19]	HUI YANG	2020	China	tissue	42	qRT-PCR	Not mentioned	2.71(1.21–6.04)	7
colorectal cancer [17]	Jing Liu	2021	China	tissue	45	qRT-PCR	Not mentioned	2.49(0.98–6.34)	6
osteosarcoma [14]	Dewei Shen	2021	China	tissue	50	qRT-PCR	median	2.94(1.13–7.67)	7

### Association between aberrant expression of EWSAT1 and OS

**Fig 2:**
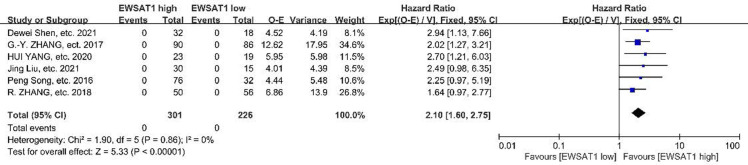
Forest plot for the association of EWSAT1 expression with overall survival.

Fig 2 presents the association between EWSAT1 aberrant Expression and OS. Of the 7 articles included, 6 articles (Peng Song, etc. 2016; G.-Y. ZHANG, etc. 2017; R. ZHANG, etc. 2018; HUI YANG, etc. 2020; Jing Liu, etc. 2021; Dewei Shen, etc. 2021) were involved in this analysis, 1 article (Iulia Virginia Iancu, etc. 2017) was excluded for not providing either HR or a Kaplan-Meier curve.

With the EWSAT1 high of 301 patients and low of 226 patients, total 527 patients, 3 articles touched the line of no effect, heterogeneity among studies was not significant (*P* = 0.86, *I*^*2*^ = 0.0%), the fixed effect model was adopted. The result showed that the overall effect estimated HR was 2.10 and 95% CI was 1.60–2.75, *P*<0.00001, which indicated high expression of EWSAT was associated with low OS. Since there was no significant heterogeneity among the studies, subgroup analysis wasn’t performed (*P* = 0.676, *I*^*2*^ = 0.0%).

### Association between aberrant expression of EWSAT1 and clinicopathological features

In order to further explore the association between aberrant expression of EWSAT1 and the prognosis of patients, several clinicopathological Features was introduced, including: metastasis (Dewei Shen, etc. 2021; G.-Y. ZHANG, etc. 2017; Iulia Virginia Iancu, etc. 2017; Jing Liu, etc. 2021; R. ZHANG, etc. 2018; n = 400), differentiation (Iulia Virginia Iancu, etc. 2017; R. ZHANG, etc. 2018; n = 129), age (G.-Y. ZHANG, etc. 2017; R. ZHANG, etc. 2018; HUI YANG, etc. 2020; n = 324) and TNM stage (G.-Y. ZHANG, etc. 2017; R. ZHANG, etc. 2018; n = 282) (Table 2).

**Table 2:** Distribution characteristics of EWSAT1 in the patients with different clinicopathological features.

**Article**	**Cancer**	**Sample size**	**metastasis**	**EWSAT1 high**	**EWSAT1 low**	**Total**
G.-Y. ZHANG 2017 [12]	osteosarcoma	176	present	55	36	91
			absent	35	50	85
Iulia Virginia Iancu 2017 [15]	cervical	23	present	3	7	10
			absent	8	5	13
R. ZHANG 2018 [16]	colorectal	106	present	26	16	42
			absent	24	40	64
Jing Liu 2021 [17]	colorectal	45	present	17	4	21
			absent	16	8	24
Dewei Shen 2021 [14]	osteosarcoma	50	present	23	6	29
			absent	9	12	21
Total (Freq): 5	4 types	400	present	124	69	193
			absent	92	115	207
**Article**	**Cancer**	**Sample size**	**Differentiation**	**EWSAT1 high**	**EWSAT1 low**	**Total**
Iulia Virginia Iancu 2017 [15]	cervical	23	well+moderate	9	7	16
			poor	2	5	7
R. ZHANG 2018 [16]	colorectal	106	well+moderate	38	34	72
			poor	12	22	34
Total (Freq): 2	2 types	129	well+moderate	47	41	88
			poor	14	27	41
**Article**	**Cancer**	**Sample size**	**Age**	**EWSAT1 high**	**EWSAT1 low**	**Total**
G.-Y. ZHANG 2017 [12]	osteosarcoma	176	<60y	47	41	88
			≥60y	43	45	88
R. ZHANG 2018 [16]	colorectal	106	<60y	38	34	72
			≥60y	12	22	34
HUI YANG 2020 [19]	glioma	42	<60y	14	9	23
			≥60y	9	10	19
Total (Freq): 3	3 types	324	<60y	99	84	183
			≥60y	64	77	141
**Article**	**Cancer**	**Sample size**	**TNM stage**	**EWSAT1 high**	**EWSAT1 low**	**Total**
G.-Y. ZHANG 2017 [12]	osteosarcoma	176	Ⅰ+Ⅱ	31	52	83
			Ⅲ	59	34	93
R. ZHANG 2018 [16]	colorectal	106	Ⅰ+Ⅱ	25	42	67
			Ⅲ	25	14	39
Total (Freq): 2	2 types	282	Ⅰ+Ⅱ	56	94	150
			Ⅲ	84	48	132

**Fig 3:**
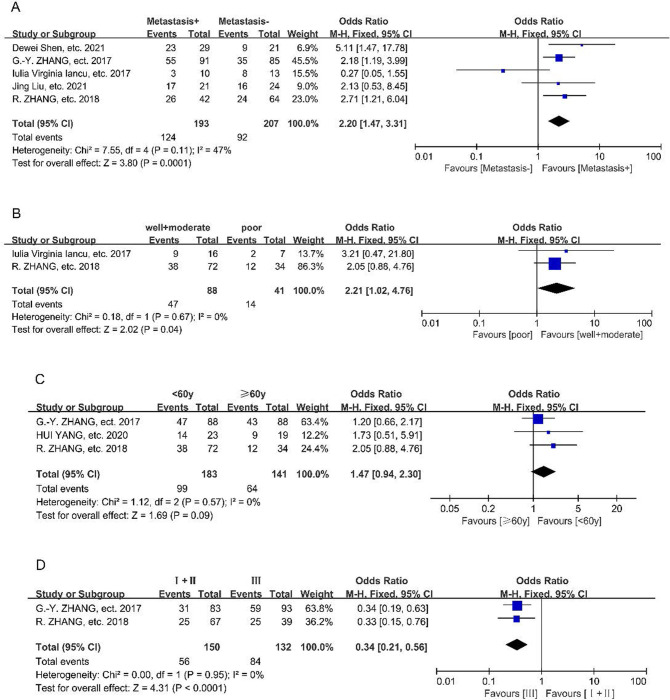
Forest plots for the association of EWSAT1 expression with several clinicopathological features. (A) Forest plots for the association of EWSAT1 expression with cancer metastasis, (B) differentiation, (C) age, (D) TNM stage.

ORs and the 95% CIs were employed to investigate the relationship between different expression of EWSAT1 and clinicopathological features above. The results were shown in Fig 3 and Table 3, which indicated that metastasis (OR: 2.20, 95% CI: 1.47–3.31, *p* = 0.0001), differentiation (OR: 2.21, 95% CI: 1.02–4.76, *p* = 0.04) and TNM stage (OR: 0.34, 95% CI: 0.21–0.56, *p*<0.0001) were associated with the aberrant expression of EWSAT1, high expression of EWSAT1 tended to have more metastasis and high TNM stage, but better differentiation. Besides, in this analysis, age (OR: 1.47, 95% CI: 0.94–2.30, *p* = 0.09) didn’t statistically show significant association with the expression of EWSAT1.

**Table 3:** Association of EWSAT1 expression with clinicopathological features.

Clinicopathological parameters studies (n)	Patients(n)	OR(95%CI)	*P*-value	Heterogeneity (*I*^2^, *P*)	Model
metastasis (present vs. absent)	400	2.20(1.47–3.31)	**0.0001**	47%, 0.11	Fixed
Differentiation grade (well+ moderate vs. poor)	129	2.21(1.02–4.76)	**0.04**	0%, 0.67	Fixed
Age (<60y vs. ≥60y)	324	1.47(0.94–2.30)	0.09	0%, 0.57	Fixed
TNM stage (I+II vs. III)	282	0.34(0.21–0.56)	**<0.0001**	0%, 0.95	Fixed

### Publication bias and sensitivity analysis

**Fig 4:**
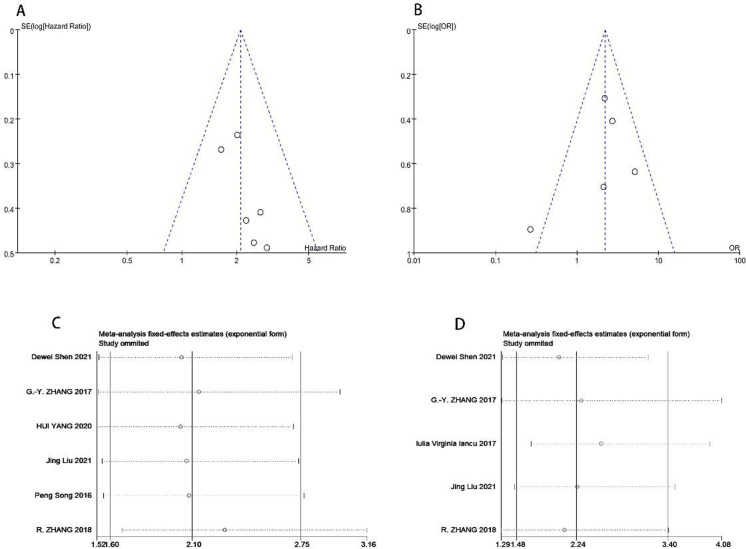
Funnel plots and sensitivity analyses. (A) Funnel plots for studies involved in analysis of HR and OS, (B) OR and metastasis;(C) Sensitivity analysis for studies involved in analysis of HR and OS, (D) OR and metastasis.

When more than three articles were involved in an analysis, publication bias was estimated by the funnel plot. Results were shown in Fig 4A and 4B. We deemed that no significant publication bias was found in the two analyses.

Sensitivity analysis was also performed in the two analyses, using local sensitivity analysis approach by excluding each eligible study one at a time. Results showed that there was no significant change in our conclusions, thereby confirmed the robustness and liability of our conclusions in this meta-analysis (Fig 4C and 4D).

### Expressions of EWSAT1 in pancancer

**Fig 5:**
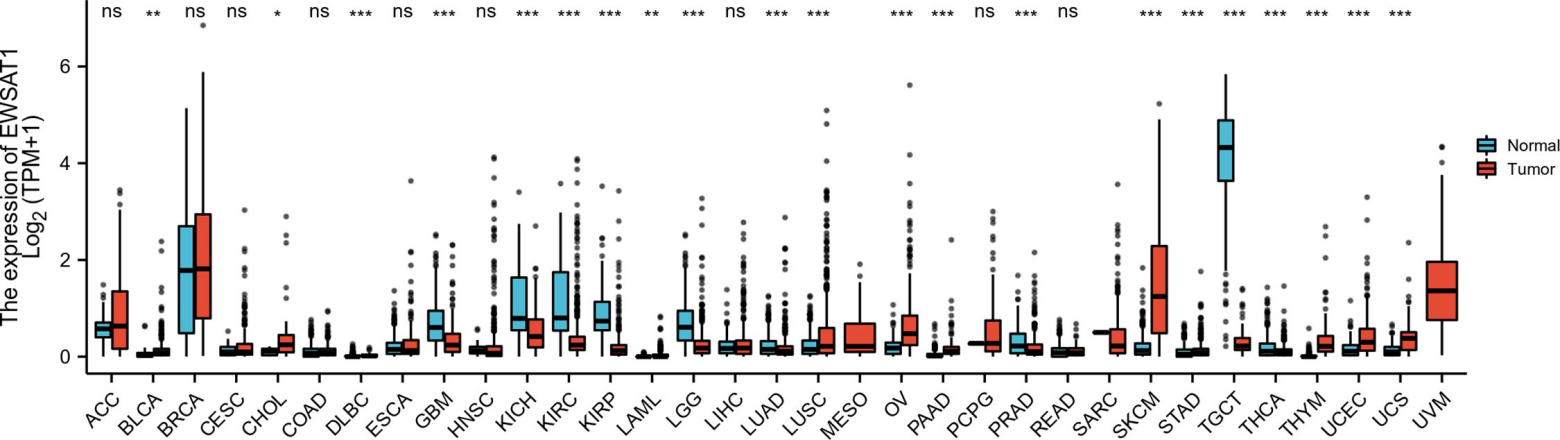
Expressions of EWSAT1 in tissues of normal and pancancer. Wilcoxon rank sum test: no significance (ns), p≥0.05; *, p< 0.05; **, p<0.01; ***, p<0.001.

The expression of EWSAT1 was explored in pancancer, results were shown in Fig 5. Aberrant expression of EWSAT1 was validated between normal and tumor tissues. Results showed that EWSAT1 was significantly increased in cancers like Skin Cutaneous Melanoma (SKCM), Glioblastoma multiforme (GBM), Ovarian serous cystadenocarcinoma (OV), and etc., while it was significantly decreased in Testicular Germ Cell Tumors (TGCT), Brain Lower Grade Glioma (LGG) and etc.

## Discussion

With the development of RNA sequencing technology, accumulating evidence shows that lncRNAs can regulate the expression of genes though many elaborate mechanism [23]. LncRNAs can react with DNA, RNA and protein directly or indirectly [24–26], and ultimately affect the function of body, resulting in dysfunctions, diseases, even cancers [27–31]. More and more studies show lncRNAs can serve as novel biomarkers or targets for diagnosis, treatment, prognosis of cancers due to their important functions in cancer generation, proliferation, progression and metastasis [8, 32–36]. Furthermore, when combined with different clinicopathological features, more accurate condition of every single patient can be got, and correspondingly more individualized treatment can be made [37, 38].

In this meta-analysis, we analyzed 6 studies with HR of OS in 4 different cancers, the overall effect showed that high expression of lncRNA EWSAT1 linked to low OS, which indicated EWSAT1 an oncogene in the cancers involved. 2 studies touched the line of no effect, but the sensitive analysis showed our conclusion was robust and reliable. Though in most studies the expression of EWSAT1 was up regulated in cancers, but there also cancers in which it was down regulated (Fig 5). This meant that EWSAT1 probably could act as a protective factor in some circumstances as well. However, constrained to the limited clinical data and studies to date, more data in this area was needed to further clarify this problem.

Moreover, the relationship between 4 clinical features and EWSAT1 expression was also investigated. We found that high expression of EWAST1 tended to have more metastasis and higher TNM stage, which usually indicated late stage of cancers and poor prognosis. This conclusion was consistent with the previous one. However, more patients of well and moderate differentiation cancers were discovered in EWSAT1 high expression group with OR 2.20, 95% CI 1.02–4.76. We would like to contribute this effect to several reasons. Firstly, the analysis included only two small sample studies (total n = 129), the sampling error was large and the result was not that stable and reliable. We call for more studies with large samples to make this conclusion robust and reliable. Secondly, more specialized subgroups were needed to further evaluate the effect. It seemed more reasonable to divide the samples into well, moderate and poor 3 groups according to the degree of differentiation. Unfortunately, no more detailed original data was available. Thirdly, the cutoff values varied in different studies, which would contribute heterogeneity in the result. Generally speaking, median was more recommended as the threshold for converting a continuous variable to a dichotomous variable in the biomedical field [39, 40]. Of course, there were also some other ways of grouping according to the data characteristics and actual needs, like mean. Although the cutoff values in the studies included were different, it’s still meaningful and of considerable value to combine these studies and make a meta analysis, because the diseases in these studies were essentially different and they were relatively independent. Last but not least, some other factors could also affect the result. For example, it’s known that some type of cancers tended to develop into high and moderate degree of differentiation, and our treatments to this type of patients were effective and mature. While the situation of poor differentiation cancers was on the opposite side, and these patients usually with short OS and small quantity that alive, which also could result in bias. In addition, Age (>60y, ≤60y) was found to have no significant association with the expression of EWSAT1 statistically in this analysis.

Aberrant expression of EWSAT1 was validated in pancancer using data from UCSC Toil RNA-seq Recompute data of TCGA and GTEx database. The aberrant expression in multiple cancers demonstrated that EWSAT1 had a strong association with varies cancers. The results of GBM and OV were in accord with the conclusions of our included studies.

In general, the results of our meta analysis suggest that lncRNA EWSAT1 acts as an oncogene in many cancers, which relate to low OS, more metastasis, higher stage of TNM stage. The expression level of EWSAT1 could be used to predict possible prognosis, imply the progression stage of cancers, indicate a cancer that may exist for early diagnosis, act as a potential biomarker and drug target in treatment. Meanwhile, when combined with several key clinicopathological features, doctors can make the most of a patient’s condition, and guide an individualized treatment.

However, some limitations of this meta-analysis should be noticed. For one thing, there’s limited data on EWSAT1 so far, only 7 eligible studies with 550 patients included, which may reduce the stringency of the conclusion. This is also suitable for analysis of the clinicopathological features. For another thing, the HRs and 95% CIs extracted by software from Kaplan-Meier curves may lead to extraneous heterogeneity. In addition, the fact that treatments varied in different cancers might affect the OS and add to heterogeneity as well. However, we deemed that it was still comparable between patients in each study and could be combined for meta analysis, for all patients underwent surgery and patients in each study were treated in the same hospital. Here, confined to the limited data and clinical fact, it’s temporarily impossible to discuss the influence of aberrant EWSAT1 expression on OS under the same treatments. Lastly, different studies or cancers have somehow different criteria for evaluating parameters and there is only one small study from non-Asian region, which may also add bias.

In summary, high expression of EWSAT1 is associate with poor OS, more metastasis, higher stage of TNM stage in many cancers. Thus, the results of our meta-analysis indicate that EWSAT1 may be selected as a potential prognosis biomarker for multiple cancers.

## Supporting information

S1 Checklist PRISMA 2009 checklist. (DOC)

## Abbreviations

EWSAT1: Ewing sarcoma-associated transcript 1
lncRNA: Long noncoding RNA
CI: Confidence interval
HR: Hazard ratio
OR: Odds ratio
OS: Overall survival
NOS: Newcastle-Ottawa Scale
SKCM: Skin Cutaneous Melanoma
GBM: Glioblastoma multiforme
OV: Ovarian serous cystadenocarcinoma
TGCT: Testicular Germ Cell Tumors
LGG: Brain Lower Grade Glioma
